# Dialytic Separation of Bundled, Functionalized Carbon Nanotubes from Carbonaceous Impurities

**DOI:** 10.3390/cryst4040450

**Published:** 2014-11-20

**Authors:** J. Justin Mulvey, Evan N. Feinberg, Michael R. McDevitt, David A. Scheinberg

**Affiliations:** 1 Molecular Pharmacology and Chemistry Program, Memorial Sloan-Kettering Cancer Center, 1275 York Avenue, New York, NY 10065, USA;; 2 Weill Cornell Medical College, 525 E 68th Street, New York, NY 10065, USA; 3 Tri-Institutional MD-PhD Program, 1230 York Avenue, 320, New York, NY 10065, USA; 4 School of Engineering, Stanford University, Stanford, CA 94305, USA; 5 Departments of Radiology and Medicine, Memorial Sloan-Kettering Cancer Center, 408 East 69th, ZRC 1941, New York, NY 10021, USA;

**Keywords:** nanotube, C_60_, dialysis, amorphous carbon, graphitic carbon, purification, functionalization, bundling

## Abstract

Separating functionalized single-wall carbon nanotubes (SWCNTs) from functionalized amorphous carbon is challenging, due to their polydispersity and similar physicochemical properties. We describe a single-step, dialytic separation method that takes advantage of the ability of heavily functionalized SWCNTs to bundle in a polar environment while maintaining their solubility. Experiments on functionalized SWCNTs were compared with functionalized, C_60_ fullerenes (buckyballs) to probe the general applicability of the method and further characterize the bundling process. This approach may simultaneously be used to purify a functionalization reaction mixture of unreacted small molecules and of residual solvents, such as dimethylformamide.

## 1. Introduction

The modification of carbon nanostructures through covalent functionalization is expanding their applicability in biomedical [1], electronic [2] and material science fields [3]. While unmodified spheroid fullerenes have been established as non-toxic in a wide variety of delivery methods, including inhalation into the lung [4], pristine separating functionalized single-wall carbon nanotubes (SWCNT) toxicity has raised concern [5,6]. Functionalization allows the attachment of fluorophores, radioisotopes, pharmaceuticals, and other ligands to carbon nanotubes and spheroid fullerenes [7–9], which, when solubilized, with variation due to adduct properties, become non-toxic [10,11], non-immunogenic [11,12], and rapidly excretable [13] substrates with a unique anisotropy and durability [14]. The intrinsic properties of the carbon nanomaterial in combination with the manner and degree in which it is functionalized allows for a vast array of potential constructs, bringing versatility to construction and application [15,16].

SWCNTs are fabricated by several methods, including High-Pressure Carbon Monoxide (HiPCO), arc discharge, and chemical vapor deposition [17]. Unlike C_60_, which can be industrially purified by sublimation, commercially available carbon nanotubes, irrespective of production method, are purchasable in sub-optimal purities, contaminated with a variety of amorphous and graphitic carbon species, as well as residual metals such as iron, nickel, cobalt, and molybdenum.

Metal catalysts and smaller soluble carbon species such as spherical fullerenes, carbon onions, and polyaromatic carbons may be removed by non-oxidative acid washes [18], sonication [19], and fritted glass filtration [20] before functionalizing the SWCNTs. Microfilters of unfunctionalized nanotubes can produce a film of “buckypaper” as high aspect ratio tubes eventually horizontally occlude the pores and pile up while contaminants readily wash through [21].

**Figure 1.**
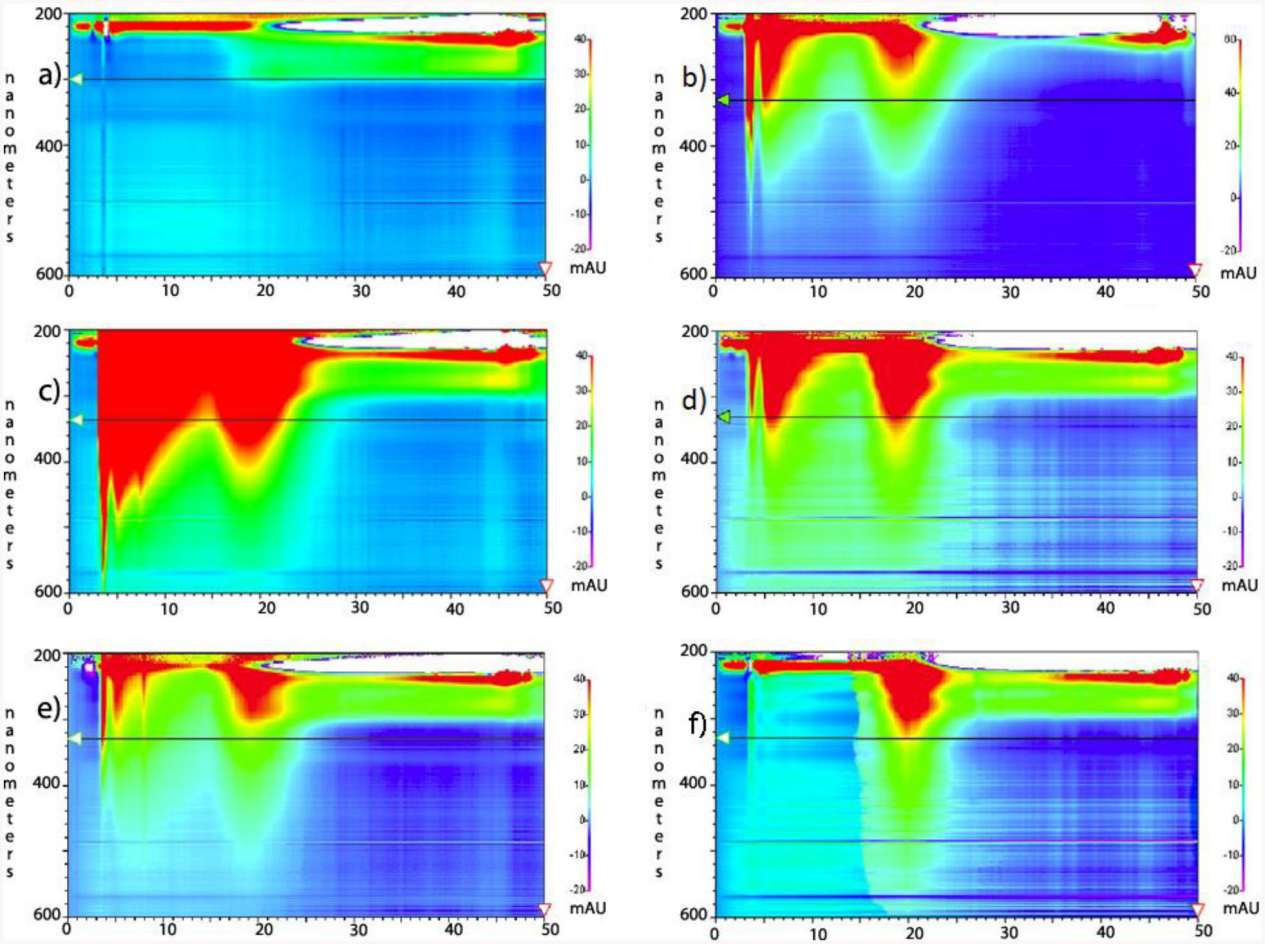
HPLC analysis of 10 k molecular weight cut-offs (MWCO) dialysis retentate and dialysate. The scale on the left *y*-axis is in the absorption wavelength in nanometers, ranging from 200 nm at the top of each chart to 600 nm at the bottom. The scale on the *x*-axis is in minutes, ranging from 0 to 50 min. Color ranging from low (purple) to high (red) represents increasing signal intensity seen on the right *y*-axis. These peaks are not quantitatively comparable and attention should be directed to the 5 min (amorphous carbon) and 20 min (SWCNT) peak ratio. (**a**) baseline wash of column; (**b**) undialyzed, functionalized SWCNTs (starting motherliquor); (**c**) dialysate Fraction #1; (**d**) retentate fraction #1; (**e**) dialysate Fraction #2; and (**f**) retentate fraction #2. The carbon peaks at 3 and 5 min in (**f**) did not diminish with subsequent rounds of dialysis and are thought to be small amounts of remaining amorphous carbon resistant to dialysis purification perhaps due to exceptional size.

However, larger contaminating carbon species are not so easily removed by these pre-functionalization exercises—and many remain even after functionalization—because these impurities often contain reactive sp^2^ carbon sectors which are functionalized along with the starting material. The products of this simultaneous functionalization of both starting materials—fullerene and carbonaceous impurity—are of similar solubility, and are not easily separated by solubility- and filtration-based separations [22,23]. Until recently, our laboratory used a short C18 column to separate the more polar modified amorphous carbon from the less polar modified carbon nanotubes [13]. The baseline gap between amorphous carbons and SWCNT peaks in C18 High-performance liquid chromatography (HPLC) analysis mirrors the preparatory potential for separation with C18 columns (Figure 1). Working with small amounts of material, columns resulted in yield loss due to adherence of product to the stationary phase. In addition, varying with species, some peaks elute closely enough to result in additional yield losses. The C18 method gave desired product in yields of approximately 18% by weight of the pre-chromatography mixture. As an alternative or supplement to C18 chromatography, the dialysis method described herein allows an economical, one-step separation of solubilized SWCNTs at a 55% higher yield.

While several methods exist to purify SWCNTs, these methods leave room for improvement in a growing need of the field: synthesis, purification, and biological use of covalently-functionalized, water-soluble SWCNTs. To summarize, while existing methods are effective at purifying unfunctionalized SWCNTs, they are minimally effective and/or low yield at purifying functionalized SWCNTs. As a result, the aim of this work is to determine a method that would purify covalently functionalized, hydrophilic SWCNTs from carbonaceous impurities in a way that is at once: (1) effective; (2) high yield; and (3) economical and accessible, and that leaves them in a bio-compatible state without the need for further purification of solvents.

The bundling of unmodified (non-functionalized) SWCNTs and the use of sonication to disperse insoluble aggregates have been well described [24]. The *in vivo* bundling of unmodified SWCNTs has been observed as aggregates in discrete organs and cell types [25,26]. Following solubilization through functionalization (Scheme S1), if SWCNTs are lyophilized to a powder [13], they require extensive sonication to resolubilize. This phenomenon is attributed to bundling properties of even functionalized SWCNTs as they engage in longitudinal pi-stacking and other non-covalent attractive interactions powerful enough to overcome the repulsive forces of sometimes ionized adducts and the entropic advantages of true solubilization.

We hypothesized that bundling of these soluble species in solution might occur when amorphous carbon and primary-amine-interacting salts were removed, thereby allowing a more pervasive, uninterrupted, alignment and packing. However, the size of these bundles would be limited because the degree of attractive tube–tube interactions on the outer rim of the bundle decreases with diameter. As a bundle increases in diameter, less surface area of each additional peripheral tube may engage in attractive interactions. Thus, a bundle’s thickness is determined by the degree of unhindered contact, of which interference and available surface area are factors.

Without the thickness of bundling, individual nanotubes, when aligned correctly by Brownian motion or with flow, may pass through dialysis membranes by a process known as fibrillary filtration, similar to glomerular filtration *in vivo* [13]. Similar phenomena have been observed with individual SWCNTs wrapped in SS-DNA that are restricted from pi-stacking and thus bundling [27].

Here we explore dialysis as a purification method for functionalized SWCNTs and C_60_.

## 2. Results and Discussion

The dialysis method, detailed here and in the Experimental section, was carried out on lysine modified SWCNTs, and buckyballs with dialysis membranes of 10,000, and 3500 dalton molecular weight cut-offs (MWCOs). Dialyses using membranes with MWCO of 20,000 and 2000 were conducted on SWCNTs only as pore-size controls, which respectively gave comparatively low yields due to loss of SWCNTs or low purity due to retention of contaminating carbon species.

After a well-characterized 1,3-cycloaddition reaction (Scheme S1) [28] SWCNTs or buckyballs at 3.00 g/L, were charged to a 2000, 3500, 10,000, or 20,000 MWCO Slide-A-Lyzer dialysis cassette (Thermo Scientific, Waltham, MA, USA). The cassette was stirred in 3 L of metal-free, deionized water (ddH_2_O, bath 1) for 30 min for the equilibration of pH and for the removal of salts. The water was then replaced with a fresh 3 L bath (bath 2). The cassette was stirred for 12 h at which point the bath turned transparent light-orange (Figure S1c). The bath 2 contents were collected, reduced to an amber oil in a roto-evaporator and lyophilized from water to a brown powder for analysis (dialysate fraction #1). The cassette retentate (which remained a deep amber in color) was sampled (retentate fraction #1), transferred to a fresh 3 L of deionized water (bath 3), and left to stir for 12 h. The now-yellow, bath 3 dialysate was collected as a brown powder (dialysate fraction #2). Dialysis of the retentate was repeated with a fourth 3 L bath of ddH_2_O. While the cassette remained amber in color, the dialysate stayed clear after 24 h (Figure S1c); no material was recovered from the dialysate. The cassette retentate was removed and lyophilized to a brown powder (retentate fraction #2). Two dialysis baths were sufficient to remove all salts and solvents; three baths were sufficient for all removable amorphous and graphitic carbon. Successful purifications have been completed in as little as 3 h per bath.

**Figure 2.**
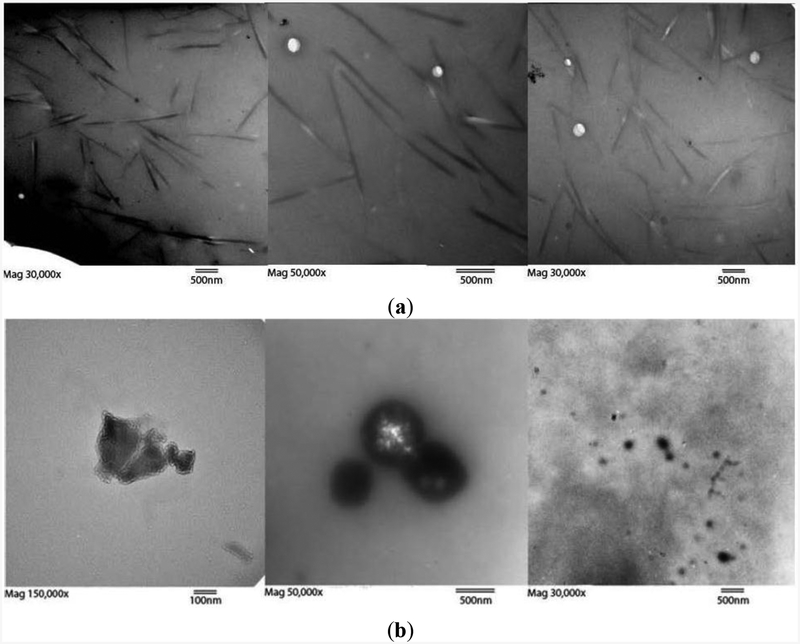
(**a**) Three TEM images of retentate after 10,000 MWCO dialysis at (left to right) magnifications of 30,000×, 50,000×, and 30,000× showing retained fractions with SWCNT bundles. White spots are electron permeable grid imperfections. Estimating a 1 nm diameter per tube, the average bundle diameter is 50 nm, or 1800 modified tubes per cross section. An enlarged image is available as Figure S2. Length bars are all 500 nm; (**b**) Three TEM images of dialysate amorphous carbon with various morphologies. No rod-like bundles were found in the dialysate, though the presence of both peaks in HPLC data suggests unbundled SWCNTs also traversed the dialysis membrane. Length bars are 100 nm, 500 nm, and 500 nm from left to right.

**Figure 3.**
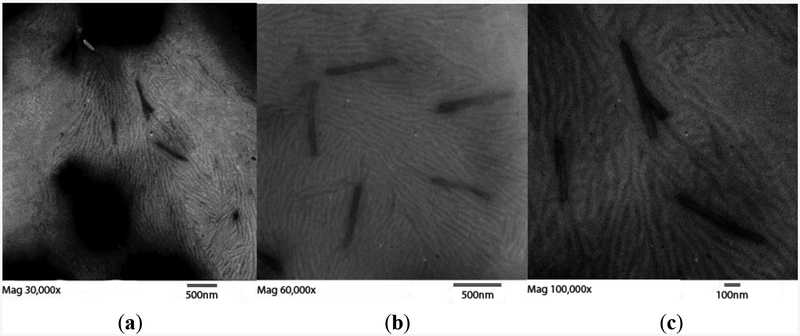
Three TEM images of retentate after 3500 MWCO membrane dialysis at magnifications of (**a**) 40,000×; (**b**) 60,000×; and (**c**) 100,000× showing retentate displaying SWCNT bundles. Two types of objects are observed. The larger bundles, as in Figure 2, are approximately 50 SWCNTs in girth. The smaller more tortuous strands seen in clusters are approximately 17 SWCNTs in girth. As seen in the zoomed-out left image these smaller bundles occur in patches, covering only a small percentage of the grid. Dark patches are out-of-focus deposits of material. Enlarged images are available as Figure S3. Length bars are 500 nm, 500 nm, and 100 nm from left to right.

Throughout the dialysis process, dialysate and retentate samples were analyzed by TEM (Figures 2 and 3), HPLC (Figure 1), and a ninhydrin Kaiser/Sarin Assay for amine content [29], representative of functionalization (Figure S1a,b). The drop in amine content per carbon from the pre-dialysis mixture to the retained, post-dialysis species (Figure S1b) reflects the greater functionalization of the removed species. This accords with HPLC analysis (Figure 1) with more polar compounds being eluted first. This more polar peak diminished with dialysis. The Kaiser/Sarin assay was also used to verify the removal of tert-butyloxycarbonyl (BOC) protecting groups during the functionalization of SWCNTs and fullerenes.

The retentates in the 3500 and 10,000 MWCO cassettes were noticeably enriched with SWCNTs and contained a paucity of amorphous carbon species compared to the dialysate when examined by TEM. The retained material was aminated (Figure S1b), fully soluble, and displayed tube bundle morphology (Figures 2 and 3). A true yield for this method of separation could not be ascertained due to the unknown original and post functionalization proportions of amorphous, graphitic and tubular carbon; however, the increased purity observed by HPLC and TEM was evident, and a yield derived strictly by weight of retentate and dialysate was 55% over our C18 chromatographic methodologies at approximately 28%, an absolute 10% increase. This separation of SWCNT bundles and amorphous carbon species did not occur with a 20,000 MWCO cassette. Instead the dialysate in the bath reached a dilute, depth-of-color equilibrium with insignificant retention.

Retentate lyophilized after this dialysis process could be re-dissolved by sonicating for 10 min in a variety of polar-protic and polar-aprotic solvents. Lyophilized dialysate dissolved immediately without need for sonication.

### 2.1. Pore Size Correlates with Functionalized SWCNT Girth Observed in Retentate by TEM

**Figure 4.**
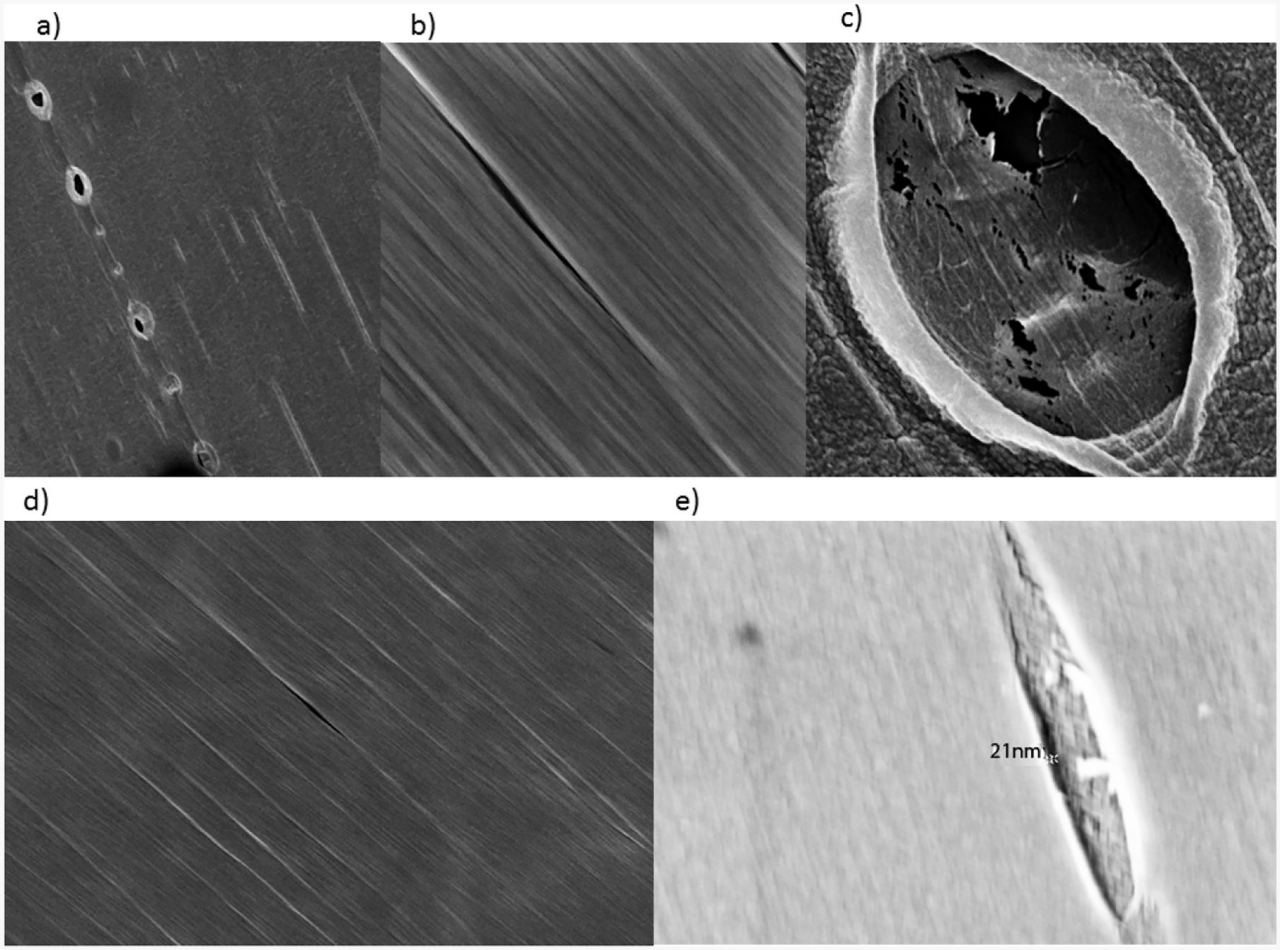
SEM images of wet, pre-dialysis cassette pores. (**Top**) 10,000 MWCO; (**Bottom**) 3500 MWCO. Pores were made by gamma irradiation of cellulose. Pore sizes identified for 10,000 MWCO range from 0 to 150 nm (**a**). The vast majority of 10,000 MWCO pores were slit-like (**b**) and sized below 40 nm. Some ellipsoid indentations (not full perforations) of questionable permeability were as broad as one micron (**c**). The pores in the 3500 MWCO were nearly allslit-like (**d**) ranging from 0 to 50 nm with the vast majority below 25 nm. Indentations (not full perforations) were seen as broad as 150 nm. The largest was taken as an extreme close up with an in-lens, the perforation within measuring ~21 nm (**e**).

TEM images of retentate from the 10,000 MWCO dialysis cassette displayed mostly long, thin bundles (Figure 2), compared to the retained material after dialysis with a 3500 MWCO membrane, which exhibited a mixture of large and small bundles (Figure 3). These bundles of functionalized SWCNTs were different in morphology from those of unfunctionalized tubes. Areas with defects leading to kinks become sites of thermally-induced oxidative cleavage in functionalization reactions allowing better formation of rods [30]. The small bundles retained in the 3500 MWCO samples were initially thought to be background, but their occurrence in patches, not covering the rest of the grid, their non-uniform arrangement, and their failure to appear in any other samples while appearing consistently in 3500 MWCO samples became the basis for a hypothesis linking pore size to bundle size. Dialysate from both MWCO sizes contained large amounts of amorphous carbon and sparse SWCNT content compared to the retentate (Figures 1–3, S2 and S3). The retention of small SWCNT bundles correlated qualitatively with the difference in pore size of the dialysis cassettes. Pores were made in cellulose by γ radiation bombardment. SEM images comparing the critical-point-dried, post-dialysis pores of a 10,000 MWCO cassette and a 3500 MWCO cassette are provided for comparison of pore size (Figure 4).

Previous studies [13] on glomerular filtration of carbon nanotubes show the ease with which a 1 nm, single, functionalized tube may pass through the glomerulus with a MWCO of 30–50 kD [31,32] even though the individual tube weight may exceed that cutoff by 10-fold. This is due to its anisotropy with aspect ratios ranging in the multi-hundreds to thousands. In the case of the kidney, the ratio of the diameter of a single SWCNT to the MWCO of the slit diaphragm is between ~0.033 and 0.02. While the described functionalized SWCNTs (Scheme S1) do not aggregate *in vivo*, they form bundles of limited size in unadulterated water. In the case of membrane dialysis *in vitro*, our modified nanotubes reached a diameter of 50 nm in a 10,000 MWCO cassette (Figures 2 and S2), thus increasing the ratio from ~0.02 to ~5.0. This is a 250-fold decrease in permissivity, and provides an explanation for the contrast of SWCNT excretion in the kidney, but retention of tube bundles in dialysis cassettes. However, another, or contributing explanation for this contrast is the charged-based selectivity of the negatively charged kidney basement membrane that supports the filtration of positively charged materials.

Methods to lysine-modify SWCNTs and C_60_ *in vivo* have demonstrated the applicability of this process as an important ancillary technique to existing purifications for producing high-purity, water-soluble, covalently-modified carbon structures [28]. When covalently lysine-modified nanotubes (SWCNT-Lys-NH_2_) were introduced to the bloodstream of mice, they rapidly cleared through the kidney’s slit diaphragm using fibrillar filtration [28]. This demonstrates that even when prepared as bundles, the environment of salts, protein, and lipids rapidly degenerated the aggregates to a point that they could quickly align with flow and are cleared via urination.

**Figure 5.**
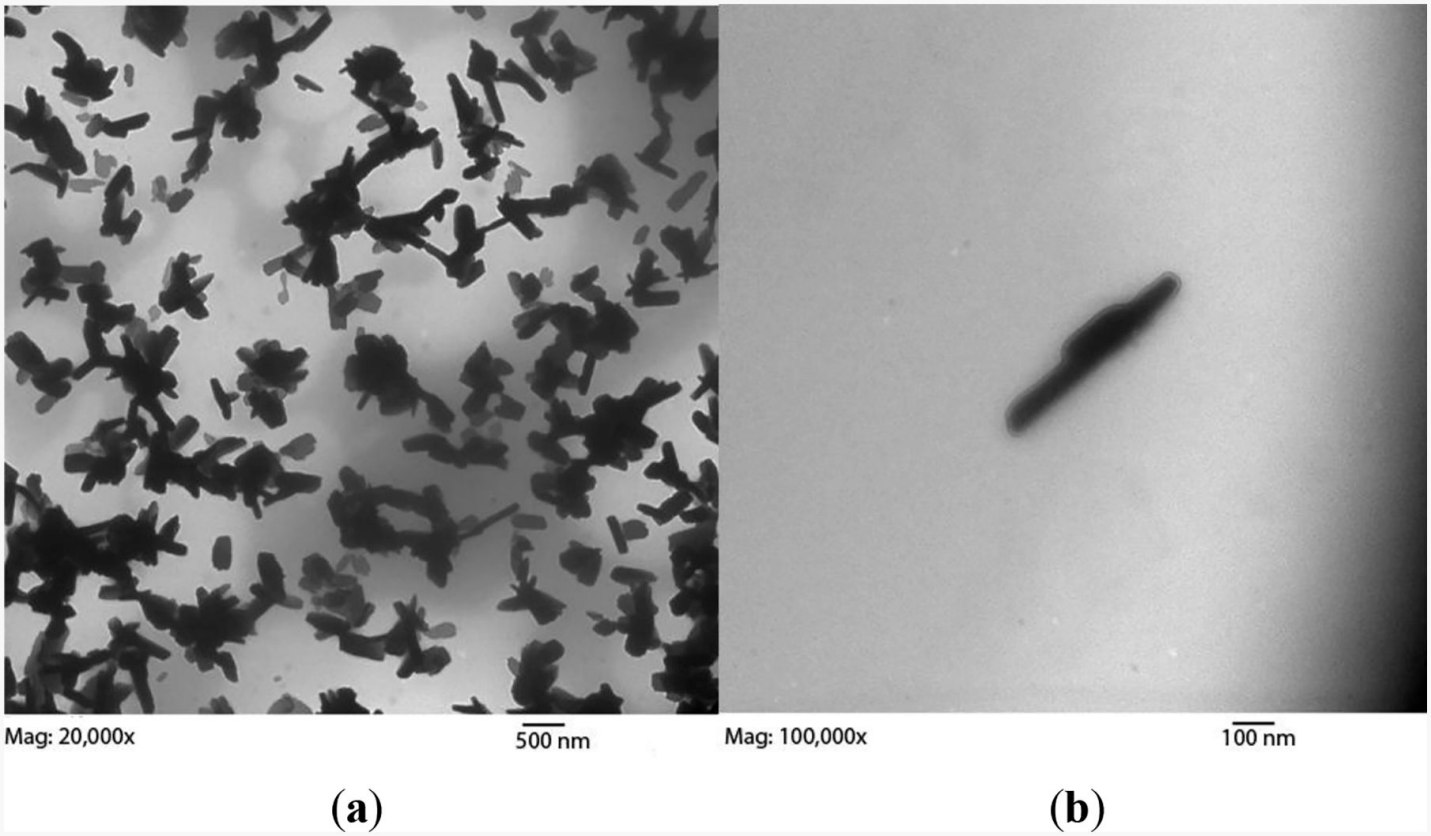
SWCNT subjected to 4 h 7 mol/L nitric acid reflux prior to functionalization and dialysis, resulting in stubbier tube bundles. (**a**) 20,000×; (**b**) 100,000×.

Deposits of amorphous carbon with diameters larger than the pores seen in Figure 4 were also found in the dialysate by TEM. These clusters of amorphous carbon are assumed to be bundled with less rigidity and thus, being amorphous by name, are able to fragment or deform, traverse the membrane, and reform in the dialysate in a short time frame, similar to what is observed with C_60_ below (Section 2.5 and Figure 5).

### 2.2. SWCNT Length Does Not Affect Bundle Girth by TEM

If the SWCNTs were allowed to reflux for 4 h in 7 mol/L nitric acid prior to functionalization, instead of allowing 10 min of room temperature stirring used in our protocol, the bundle length shortened, as the long-lived oxidative conditions divided the SWCNTs into smaller and smaller pieces at oxidizable sites of imperfection (Figure 5). 7 mol/L nitric acid treatments over variable times is an effective way to control SWCNT length [33]; however, the bundle diameter was similar to that of longer bundles found in the 10 min, room temperature protocol. Non-destructive purification methods may be employed to prevent changes in morphology [33,34].

### 2.3. Sonication Disrupts Bundles and Allows Membrane Passage of SWCNTs

A proof of concept bundling experiment was conducted by removing a terminally dialyzed cassette of functionalized SWCNTs that had not (after 24 h) noticeably changed the color of a fresh 1 L water bath, similar to the picture shown (Figure S1c). This cassette was then sonicated for 10 min with the intention of disrupting the bundles [24] before being returned to the same beaker of ddH_2_O. The liquid color changed to faint yellow in 30 min, but did not progress beyond this, and did not reach an equilibrium, as evidenced by color change, with the contents of the cassette. This is hypothesized to reflect a debundling with a scant release of material, before a rebundling of cassette contents. It also sets a timeframe for the bundling process of our lysine-functionalized tubes. The procedure was then repeated using the same cassette and bath causing dialysate to become a darker yellow, again ceasing progression shortly after. Ultraviolet/visible measurements corroborated the visual findings showing an absorbance increase in the dialysate with each subsequent sonication, but with a smaller increase on each iteration. These results demonstrate that the permissivity of SWCNTs after the first sonication did not stop because of an energetic or concentration-based equilibrium with unbundled SWCNTs, but rather because the SWCNTs debundled when sonicated and were allowed to traverse the membrane with their reduced girth. The nanotubes with less functionalization should have stronger pi-stacking interactions and should bundle faster. Thus, the functionalized SWCNTs lost with each progressive sonication are hypothesized to be the more functionalized tubes of the bundle. This technique could be used to fine-tune the desired degree of SWCNT functionalization.

In a similar experiment, when lyophilized dialysate that was not sonicated was put into a fresh dialysis cassette and allowed to dialyze again in fresh ddH_2_O, it reached a full equilibrium with the water in 2 h, emphasizing that the amorphous and graphitic carbon contained in the dialysate were unable to bundle to a degree that would prevent them from passing through the membrane.

### 2.4. Bundle Packing Computational Analysis

**Figure 6.**
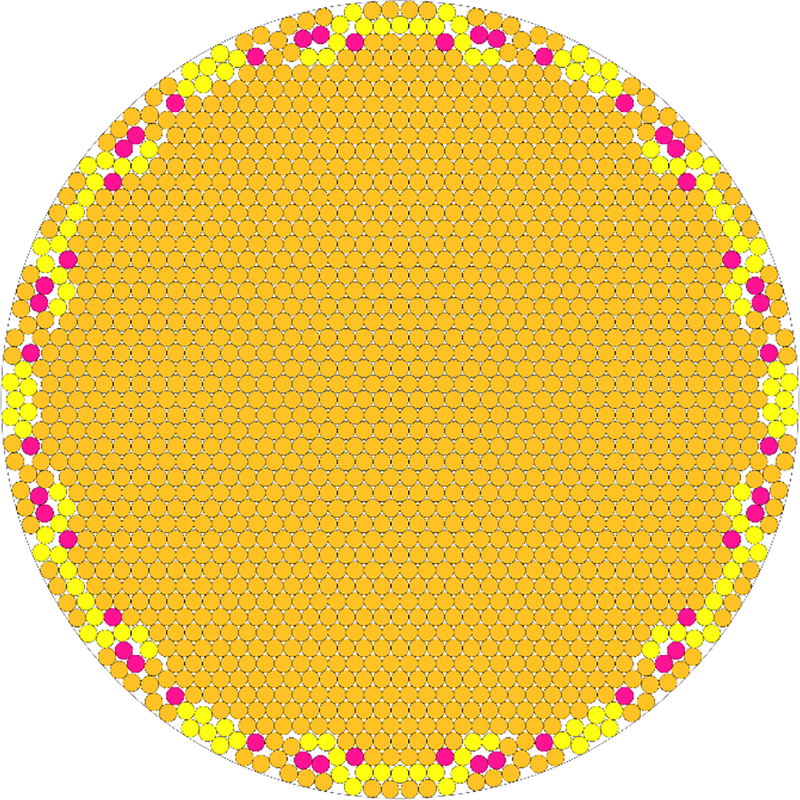
Code output derived cartoon showing optimal packing of 1735 circles of equal radius in a larger circle. Yellow and pink colored circles correspond to predicted loosely packing nanotubes in the outermost layers of the bundle as bundling energy is decreased with available surface area.

To better characterize the hypothesis that carbon nanotube bundling precluded its elution into the dialysate, numerical experiments were conducted to predict bundle content. The 10,000 MWCO cassettes seemed to retain bundles of approximately 50 nm in diameter, and the 3000 MWCO of 17 nm in diameter at room temperature. While we do not expect these girths to be discreet, they were used as examples in the following analysis. Given that a carbon nanotube has a diameter of approximately 1 nm, numerical experiments were conducted to estimate the number of nanotubes in a given bundle. Computing such an estimate falls under circle packing, a classic mathematical problem for which few analytical solutions exist. Therefore, a specialized numerical optimization code was used to calculate the number of circles of radius 1 nm that can fit in a larger circle of radius 50 nm, which was then repeated for a larger circle of radius 17 nm. An estimate can be found with the equation:

(1) $$N={\text{e}}^{\frac{\text{ln}\left(r\right)-\text{ln}\left(0.83504\right)}{-0.48397}}$$

where *N* is the number of circles that can fit in a larger circle with a radius ratio of 1/*r*. Therefore, for our problem *r* = 0.02 (for the 50 nm bundle) and *r* = 0.0588 (for 17 nm bundle), yielding 2232 nanotubes for the former and 240 nanotubes for the latter case. This estimate corresponds well to the results of a computational experiment, which predicts between 2100 and 2200 nanotubes for the 50 nm bundle and between 243 and 244 for the 17 nm bundle. Due to the lysine side chains adducted to each nanotube, an important caveat of this analysis is that the “effective radius” of each individual nanotube may in fact be larger. This, however, does not affect the empirically derived bundle girth. Since each carbon-carbon bond has a length of approximately 1.53 Å, we can estimate the effective diameter of a lysine functionalized nanotube to be between 1.5 and 2.0 nm. This reduces the estimate for the 50 nm bundle to be between 1200 and 1400 nanotubes, and the estimate for the 17 nm bundle to be between 50 and 60 nanotubes. This assumes a uniform distribution of adduct on the nanotube, which the field has not yet established for SWCNT additions in general. A model of the sample output of the program is seen below. The model assumes the geometric ideal of tessellating hexagons. However, in practice each nanotube has a different length and level of modification. This renders packing quasi-hexagonal at best, with increasing non-uniformity as bundle radius increases. As a result, the outer layer is the least hexagonal, geometrically rougher and higher energy due to non-ideal alignment. This circle packing model does not account for the variable placement and spacing of adducts on individual nanotubes, nor does it account for their imperfections such as Stone-Wales transformations that might leave them kinked. Points of sloping and dysalignment will exist and any cross section like that shown in Figure 6 will not be uniform. This compounding disunity, layer by layer, is one possible explanation as to why the bundles do not grow to be large visible structures that would potentially precipitate out of solution.

### 2.5. Functionalized C_60_ Buckyballs Bundle in Clusters and May Also Be Dialyzed

Similarly lysine-modified C_60_ buckyballs, were retained impermanently by the 10,000 MWCO cassettes for 72 h in ddH_2_O, and were retained by the 3500 MWCO cassettes for 240 h. Impermanent retention is defined here as the retentate, which started with 100% of the functionalized C_60_, remaining at least 250% of the concentration of the dialysate. This value does not encompass osmosis, which is also a factor in reducing the concentration of the retentate. Concentration was tracked with ultraviolet/visible absorbance and visualized by TEM (Figure S4). C_60_ is purchased at 99.9% purity, thus no amorphous or graphitic carbon is expected post modification, and yet the buckyball was not dialyzing freely like a small molecule of molecular weight below 1000 daltons and a maximum diameter of 2–3 nm. TEM images show a clustering, cloud-like aggregation of the modified buckyball molecules in both the dialysate and retentate. We hypothesize that this is a similar, though due to the shape of the C_60_, less strong, bundling to that of the functionalized SWCNTs. This clustering is responsible for the slow equilibration of the retentate with the dialysate of a molecule of only 835 daltons. These images are similar to published TEM images of unmodified C_60_ in polar protic solutions [35]. Despite their theoretical inability to aggregate as strongly as SWCNTs due to their limited contact surface area, functionalized buckyballs resisted transit to the dialysate. This finding allows for limited dialysis to be conducted on modified, water-soluble, C_60_ for the rapid removal of unwanted solvents, reaction materials, or salts. After 24 h in a 3500 MWCO cassette 92% of the initially charged functionalized C_60_ remained in the cassette as measured by dry weight.

### 2.6. Dialysis in More Dispersive Solvents Fails

It was noted when a post-rotovapping, soluble nanotube oil was less pre-diluted with water—that is, a higher ratio of residual DMF to water was present before charging the solution to the cassette—the faster the dialysis occurred. Concern arose that the DMF was dissolving the Slide-A-Lyzer dialysis membrane, but exposure of the membrane to a 1:1 mixture of DMF and water for one hour before rinsing and starting the dialysis process in water with aqueous SWNT-Lys-NH_2_ had no apparent effect on the outcome. It was hypothesized, in line with our general hypothesis, that the presence of DMF disrupted the energy benefit of bundling. An experiment was performed in which a 1 L dialysis bath was filled with a 1:1 mixture of DMF and water, and another with a 1:1 mixture of *N*-methylpyrrolidinone and water. Nanotubes solubilized only in water were charged to a 3500 MWCO dialysis cassette and allowed to dialyze to completion in a series of water baths. After this the cassette was charged to either of the 1:1 mixture baths. Within 4 h, the cassette and the bath had reached an equilibrium, indicating that the presence of either DMF or *N*-methylpyrrolidinone engenders further emission of nanotubes from the dialysis cassette than permitted by water alone. This makes an argument against a physical phenomenon—akin to the unmodified nanotubes in [21]—wherein a build-up of orthogonally staked tubes on the dialysis membrane pore causes a gradual ceasing of dialysis. In contrast, it supports nanotube bundling as the mechanism behind the dialysis-based purification described in this study, and supports hydrophobicity as one of the driving forces for the bundling itself. This perhaps limits the solvent range in which dialysis is possible.

## 3. Experimental Section

All reagents were obtained from Sigma-Aldrich. Modification reactions performed on C_60_ were equivalent to those on SWCNTs with the exception of temperature reaching only 115 °C; the comparative steric strain of C_60_’s enable cycloaddition to occur in milder reaction conditions.

### 3.1. Nanotube Preparation

#### 3.1.1. Nanotube Pre-Functionalization Purification

HiPCO fabricated SWCNTs with purity of ~90% carbon species and ~70% SWCNTs; 0.7–1.3 nm in diameter with a mode length of 800 nm (Sigma-Aldrich, St. Louis, MO, USA) (Figure S5) were washed with distilled water over a glass frit, and charged to a round bottom flask. To this flask was added 7 mol/L nitric acid in a submerging excess, and the dispersion was stirred for 10 min at room temperature. The contents were quenched with a 0.01 M sodium bicarbonate solution until bubbling was no longer apparent. The dispersion was then poured over a glass frit and washed 3× with dH_2_O and dried under vacuum with a Büchi RE 111 Cold Finger Rotary Evaporator (Büchi, Flawil, Switzerland).

#### 3.1.2. Nanotube Functionalization

The dried material was charged to a round bottom flask and dispersed in DMF, sonicated for 10 min, stirred, and brought to 130 °C. Over four days were added paraformaldehyde and H–Lys(BOC)–OH (CAS 2418-95-3), a side-chain protected amino acid (Scheme S1) in a 1:2:2 molar ratio (based on the moles of carbon by weight of the as purchased SWCNTs). The mother liquor gradually turned from a clear liquid with dispersed black SWCNTs to an amber solution of fully soluble SWCNTs. The reaction mixture was filtered over a glass frit to remove sediment and then through a Millipore PTFE filter (45 μm pore size, Billerica, MA, USA) to remove smaller particulate. To the filtrate was added an excess of trifluoroacetic acid (TFA) for removal of BOC groups. The solution was shaken for 15 min, and quenched with 0.1 M sodium bicarbonate to pH 9 before being charged to a Slide-A-Lyzer dialysis cassette (Thermo Scientific, Waltham, MA, USA).

### 3.2. Nanotube Dialysis

The functionalized SWCNTs were transferred to either a 2000, 3500, 10,000 or 20,000 dalton molecular weight cut-off (MWCO) (Slide-A-Lyzer) dialysis cassette. The cassette was stirred in 3 L of ddH_2_O (distilled, deionized water) from a PureLab Ultra Water Purification System (Woodridge, IL, USA). After 30 min to normalize pH and desalt the functionalized SWCNTs, the dialysis cassette was moved to a second 3 L beaker of ddH_2_O. The cassette was left to stir for 12 h turning the dialysate a transparent orange. Dialysate was collected, rotary evaporated and lyophilized to a brown powder. The dialysis cassette was moved to a third 3 L beaker of ddH_2_O and left to stir for 12 h turning the dialysate a transparent light-yellow. Dialysate was again collected, rotary evaporated and lyophilized to a brown powder. The cassette was moved to a fourth 3 L beaker of ddH_2_O, but after 24 h there was no color change to the dialysate, nor could any material be recovered via evaporation. The retentate was extracted from the cassette, and lyophilized to a brown powder.

### 3.3. Methods for Characterization

#### 3.3.1. HPLC

HPLC analysis was performed using a Beckman-Coulter System Gold Bioessential 125/168 diode array detection system (Fullerton, CA, USA), and materials were run through a C18 column using a 0.1% Trifluoroacetic acid in water/acetonitrile solvent system.

#### 3.3.2. Amine to Carbon Ratios

Amine to carbon ratio calculations were performed using a Spectramax M2 from Molecular Devices (Sunnyvale, CA, USA). Lyophilized functionalized SWCNTs were diluted to specific molalities to create a concentration curve based on absorbance at 600 nm wavelength. This curve was then used to determine the grams per liter of carbon nanotubes assuming functionalization to be of comparatively negligible weight. Molar concentrations of fullerene carbon were derived by dividing the concentration in (g/L) by 12 g/mol to yield (mol/L). A Kaiser/Sarin assay for detecting primary amines was then performed on the same sample, giving molar concentration of amines present. Dividing these two values gave primary amines per carbon.

#### 3.3.3. Transmission Electron Microscopy

TEM analysis was conducted on a 1200EX Transmission Electron Microscope (JEOL, Tokyo, Japan). Selected images are representative, and were obtained by Nina Lampen of the MSKCC Electron Microscopy Core Facility. Sample preparation was performed by spotting 5 μL of solutions onto either copper or carbon 200 mesh grids with either rapid high-vac removal of solvent or dehydration. A comparison method of flash freezing the drop with liquid nitrogen on the grid and then allowing it to sublime on high-vac showed identical results.

#### 3.3.4. Scanning Electron Microscopy

Water saturated cellulose membranes from Slide-A-Lyzer dialysis cassettes were sectioned and subjected to supercritical drying on a custom built system used in the Memorial Sloan Kettering Electron Microscopy Core (New York, NY, USA) in order to preserve the hydrated pore size. The samples were subsequently imaged on a Zeiss Supra 25 SEM with a GEMINI in-lens detector (Zeiss, Oberkochen, Germany).

### 3.4. Bundle Content Calculations (Based on Diameters Derived from 10 kD MWCO TEM Images)

Determining the number of carbon nanotubes in a larger bundle of carbon nanotubes can be treated by analogy to the circle packing in a circle problem. In finding the maximum number of congruent circles that can be completely encompassed by another circle of larger radius without overlap, relatively few analytical solutions exist. Rather, one can use various numerical optimization schemes to solve the problem. One such code was developed by Eckhardt Specht, who computed additional circle packings relevant to the nanotube study. The code uses a threshold algorithm with iterative improvement [36], and mimics a thermodynamic system. The packings have since been published online [37].

## 4. Conclusions

Dialysis of a SWCNT functionalization reaction mixture, allowing the gradual removal of interference by salts, solvents, and amorphous carbon, leads to pi-stacking with greater order and alignment, as well as van der Waals and other non-covalent nanotube-nanotube affinities. In bundling, the nanotubes collectively increase in girth and are unable to traverse dialysis membrane pores that they would easily fit through on their own when aligned longitudinally. Other explanations such as the occlusion of dialysis membrane pores by orthogonally aligned nanotubes as seen with “buckypaper” were considered, but these explanations are unappealing. The full solubility of the material, which suggests against static aggregation, the fact that smaller length nanotubes are equally well retained, the realization that buckyballs also resist dialysis in a way that similarly weighted molecules do not, and the fact that the presence of debundling solvents such as a 1:1 mixture of water and DMF lead to a rapid and complete dialysis together support the formation of nanotube bundling as the primary mechanism for the dialysis-based purification. The energetics of limited aggregation leading to the formation of bundles large enough to resist dialysis, but not so large that those bundles precipitate out of solution are complex. In an aqueous solution, factors including bundle surface area, packing heterogeneity, van der Waals forces, hydrophobicity, entropy, π–π interactions, and ionic forces contribute to this observation.

This method of purifying functionalized SWCNTs from carbonaceous impurities demonstrates utility in efficient and economical preparation for a variety of applications. Dialysis of nanotubes can separate carbonaceous impurities simultaneously with reactants and biproducts from functionalization reactions, and, at some pore sizes, and in limited time frames, dialysis can even be used to purify functionalized spheroid fullerenes. This purification method has already proven its utility in the subsequent research of our laboratory to produce bio-compatible agents of high purity for use *in vivo*.

## Supplementary Material

1
