# Supplementary material for: Dialytic Separation of Bundled, Functionalized Carbon Nanotubes from Carbonaceous Impurities
Source: Crystals (Basel). Author manuscript; Available in PMC 2021 May 11. (PMC8112586; doi:10.3390/cryst4040450)

Supplementary Information

(1) The first supplementary figure is an analysis of amine content on the dialyzed product and two pictures illustrating the dialysis process.
(2) The second supplementary figure depicts the unadultered SWCNTs as purchased from Sigma-Aldrich before being used in these experiments.
(3) The third supplementary figure depicts clusters of functionalized Buckyballs before and after dialysis. As there is only one carbon form present, they are identical as expected.
(4) The fourth supplementary figure is an enlargement of an aspect in Figure 2 for better gauging SWCNT bundle diameter from a 10,000 MWCO dialysis cassette.
(5) The fifth supplementary figure is an enlargement of an aspect in Figure 3 for better gauging SWCNT bundle diameter from a 3500 MWCO dialysis cassette.
(6) The First Supplementary Scheme illustrates one of the prominent functionalization reactions used in these experiments, the Prato reaction.

Scheme S1. Synthesis of SWCNT-Lys-NH_2: A 1,3-lysine based cyclo-addition functionalization of SWCNTs using azomethine ylide chemistry.

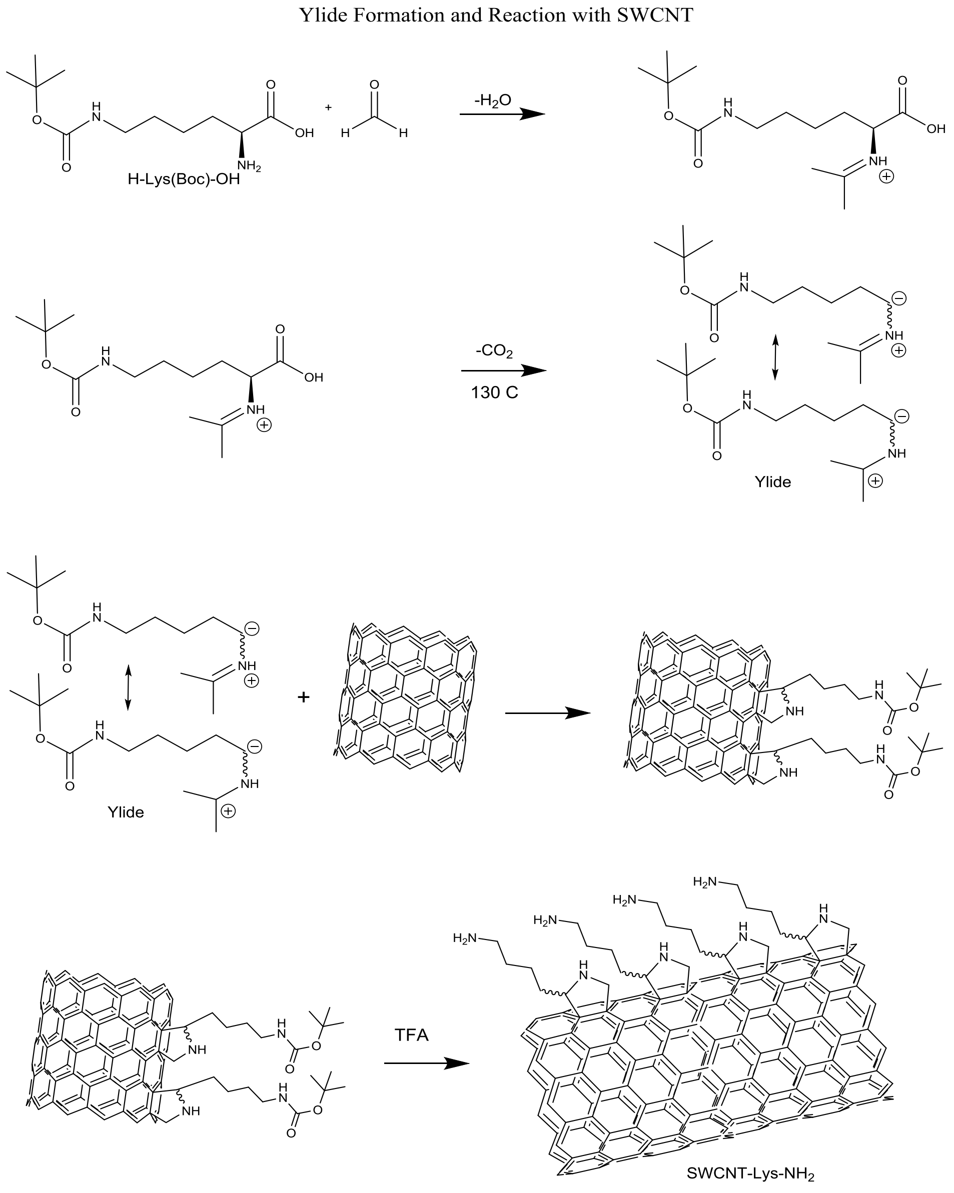

Figure S1. Pre and Post dialysis amine content. Amine to carbon ratios based on exact, lyophilized gram amounts, not concentration curves. (**a**) Kaiser assay amine content standard curve used in this experimen; (**b**) Amine to carbon ratios of representative species used in these experiments; (**c**) Photo (left) of 10,000 MW cutoff after 4th dialysis—depicting clear dialysate—and photo (right) of 3500 MW cutoff after 1st dialysis—depicting orange dialysate.

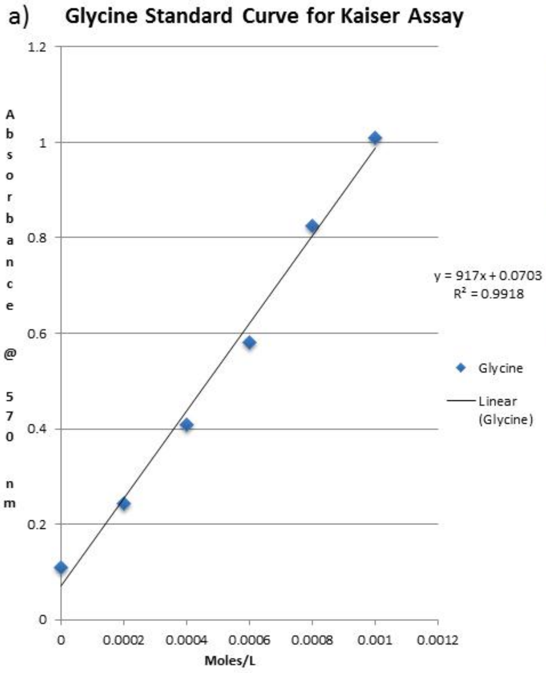

b) **Amine Content in 1 Amine per x Carbons**

Carbon Structure	Pre-Dialysis Amine Content	Post-Dialysis Amine Content
SWCNTs 10k	70.6	132.1
SWCNT 3.5k	70.6	121.8
Ribbons 10K	320.2	581.7

c) **Photo of soluble SWCNTs in 10,000 MWCO cassette after 3rd dialysis. The SWCNTs are retained by the cassette in a water bath**

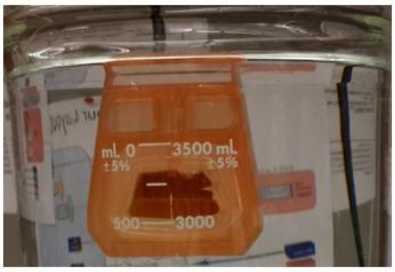

To determine the amine content of purified compounds, the ninhydrin-based Kaiser Assay was employed. Each purified compound was first frozen and lyophilized. Subsequently, concentration curves for each sample were made by measuring absorbance at A600 versus substance concentration in grams per liter. Interpolating from a standard glycine curve at A570, we determined the respective amine concentrations of each material. The concentration of nanotube carbon was estimated by dividing the grams/liter concentration by the molar mass of carbon, since carbon from the nanotubes themselves far outweigh contribution from the carbon contained in attached moieties. This procedure yielded mol/L figures for both [R-NH_2] and for [carbon from SWNT].

© 2014 by the authors; licensee MDPI, Basel, Switzerland. This article is an open access article distributed under the terms and conditions of the Creative Commons Attribution license (http://creativecommons.org/licenses/by/4.0/).

Figure S2. Englarged TEM images of functionalized SWCNT retentate after 10 kD MWCO dialysis for better bundle diameter assessment.

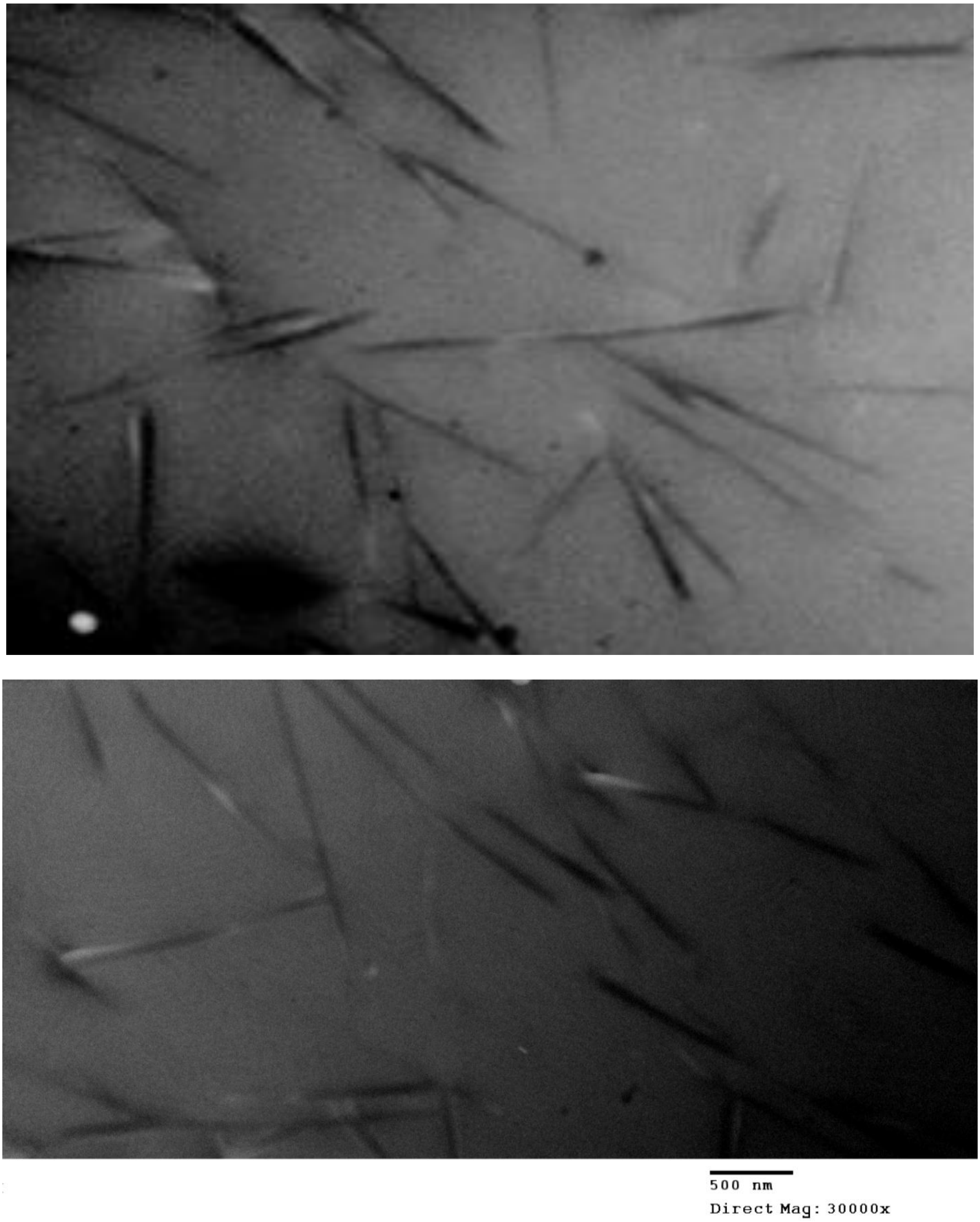

Figure S3. SWCNT bundles prior to purification or functionalization.

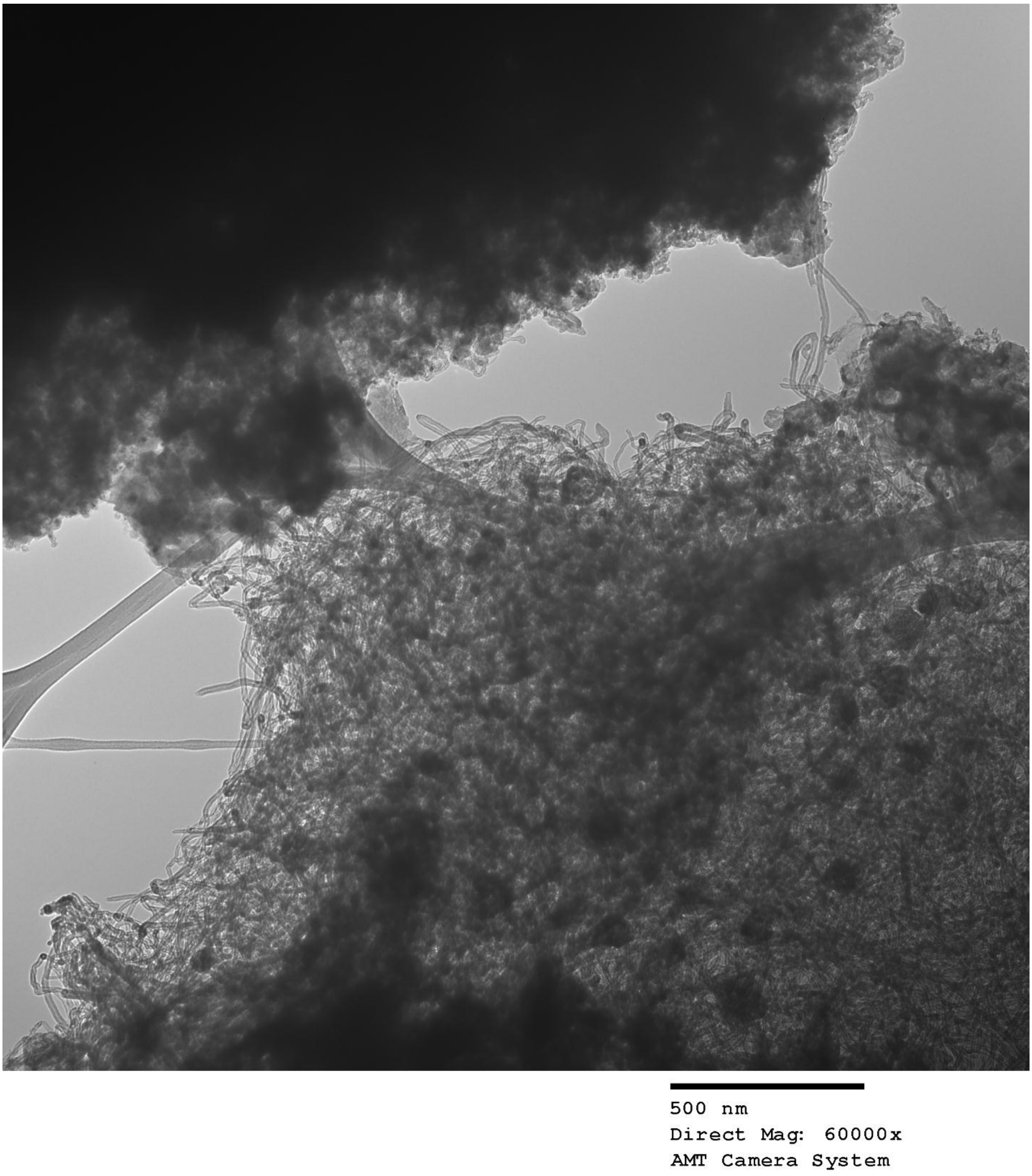

Figure S4. Functionalized buckyball clusters after 10,000 MWCO dialysis; retained material (**a**) 150,000 × and dialysate (**b**) 75,000 ×.

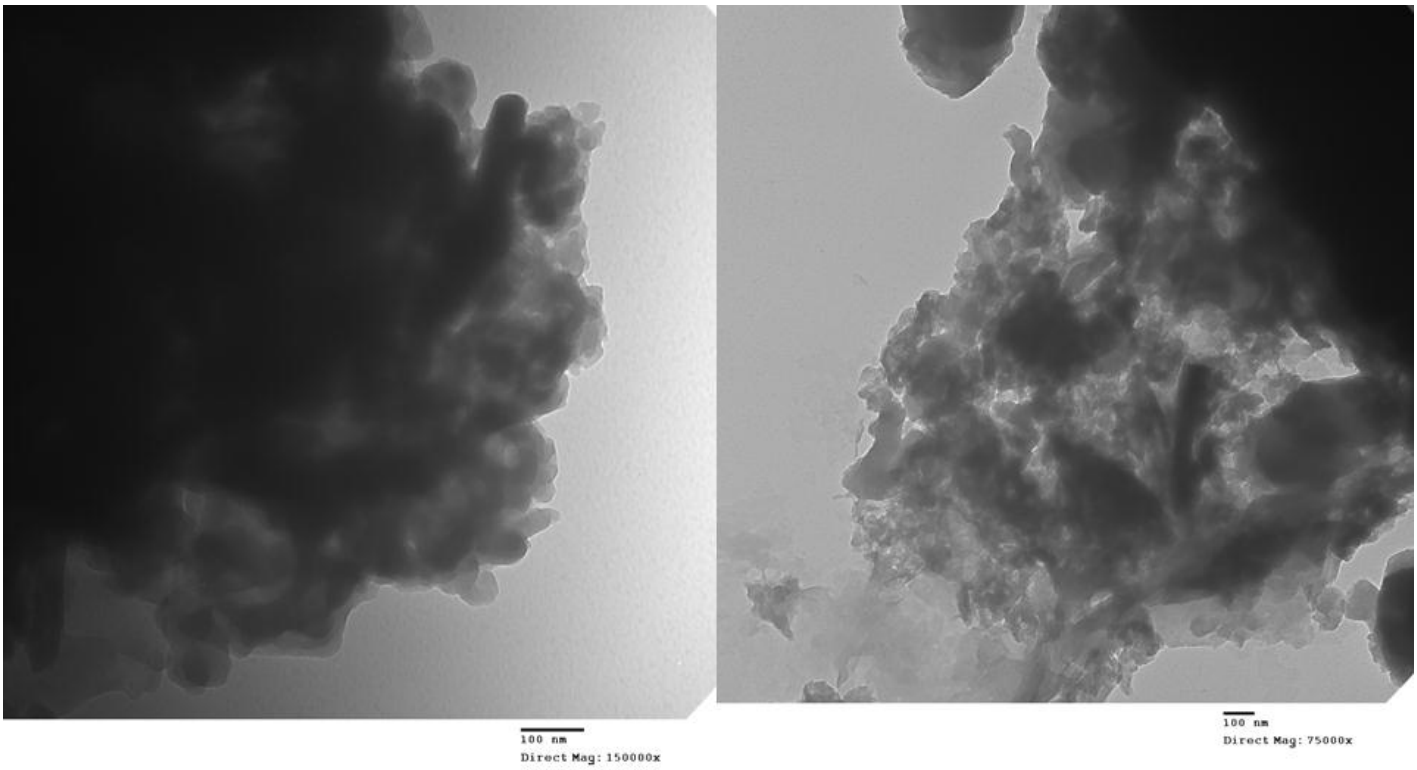

Figure S5. Englarged 3500 MWCO retentate of functionalized SWCNTs for better bundle diameter assessment.

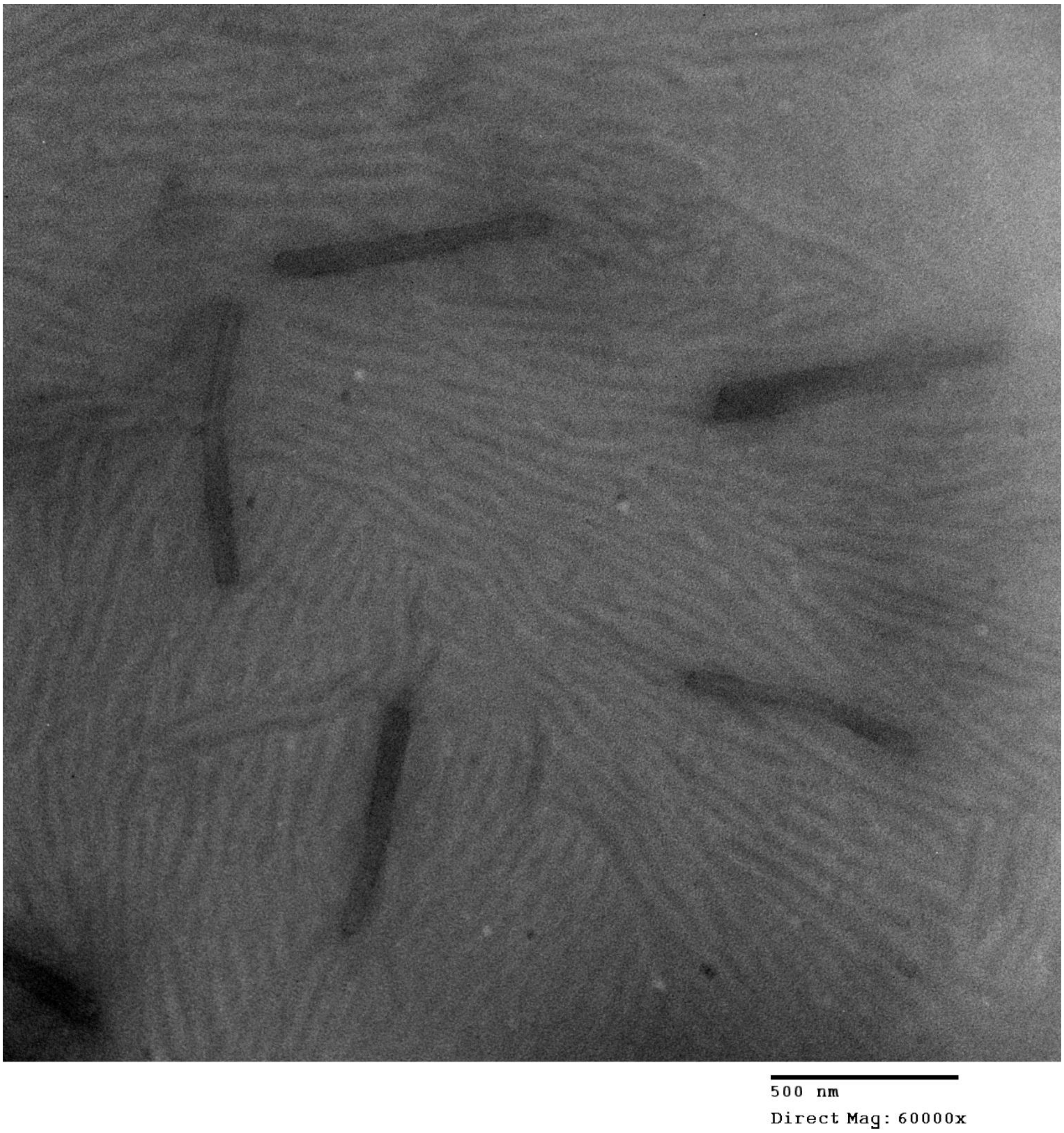

Figure S5. *Cont.*

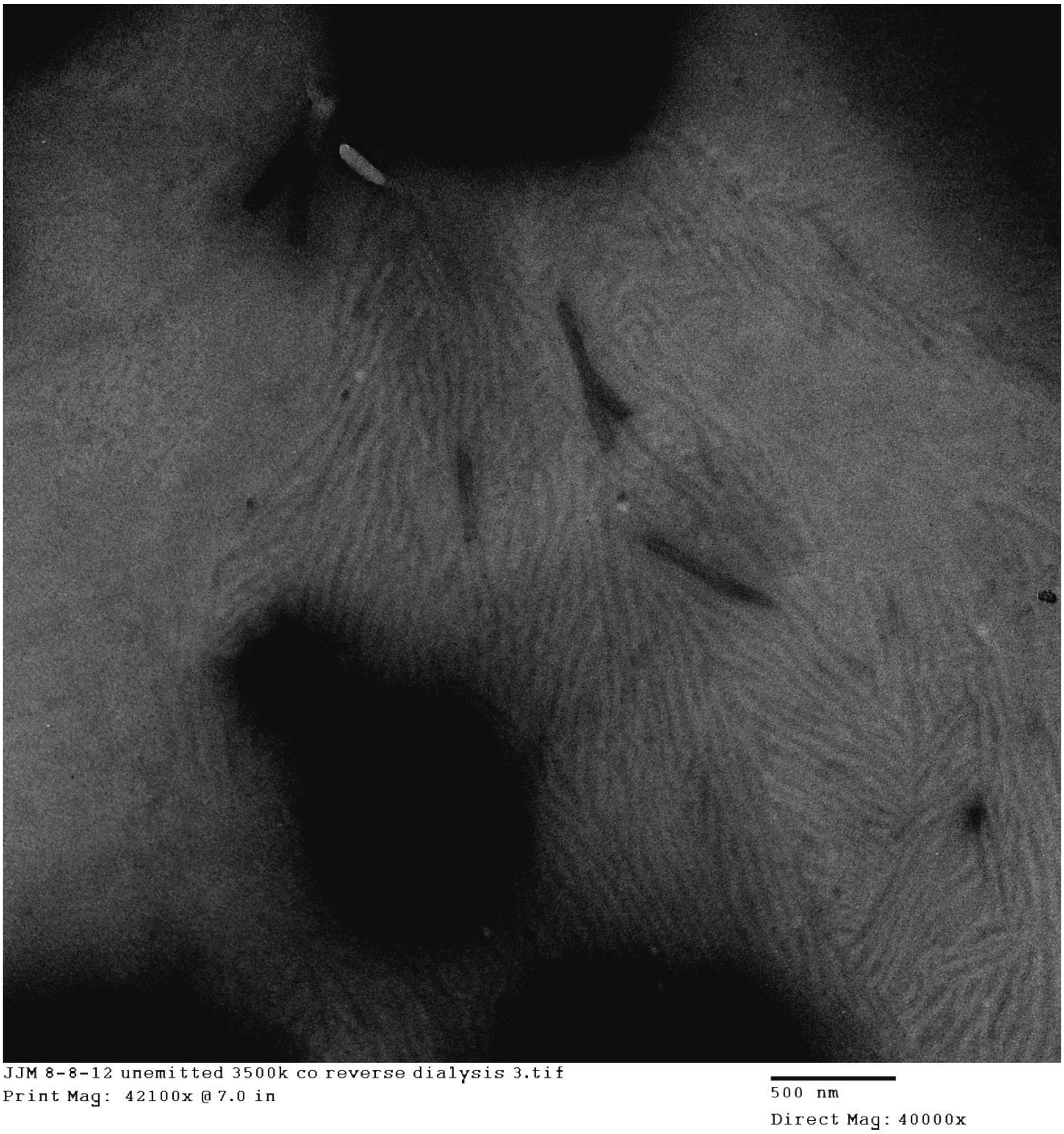

Supplement: 1 [file NIHMS1062601-supplement-1.pdf]
